# Targeting lipid droplet lysophosphatidylcholine for cisplatin chemotherapy

**DOI:** 10.1111/jcmm.15218

**Published:** 2020-06-16

**Authors:** Lumin Chen, Wen‐Lung Ma, Wei‐Chung Cheng, Juan‐Cheng Yang, Hsiao‐Ching Wang, Yu‐Ting Su, Azaj Ahmad, Yao‐Ching Hung, Wei‐Chun Chang

**Affiliations:** ^1^ Department of OBS & GYN BenQ Medical Center Suzhou China; ^2^ Department of OBS & GYN Sex Hormone Research Center Research Center for Tumor Medicine Chinese Medicine Research and Development Center China Medical University Hospital Taichung Taiwan; ^3^ Graduate Institute of Biomedical Sciences Graduate Institution of Cancer Biology Graduate Institute of Public Health China Medical University Taichung Taiwan; ^4^ Department of Nursing Asia University Taichung Taiwan

**Keywords:** lipid droplet, low‐density lipoprotein receptor, lysophosphatidylcholine, platinum‐based chemotherapy

## Abstract

This study aims to explore lipidic mechanism towards low‐density lipoprotein receptor (LDLR)‐mediated platinum chemotherapy resistance. By using the lipid profiling technology, LDLR knockdown was found to increase lysosomal lipids and decrease membranous lipid levels in EOC cells. LDLR knockdown also down‐regulated ether‐linked phosphatidylethanolamine (PE‐O, lysosomes or peroxisomes) and up‐regulated lysophosphatidylcholine [LPC, lipid droplet (LD)]. This implies that the manner of using Lands cycle (conversion of lysophospholipids) for LDs might affect cisplatin sensitivity. The bioinformatics analyses illustrated that LDLR‐related lipid entry into LD, rather than an endogenous lipid resource (eg Kennedy pathway), controls the EOC prognosis of platinum chemotherapy patients. Moreover, LDLR knockdown increased the number of platinum‐DNA adducts and reduced the LD platinum amount. By using a manufactured LPC‐liposome‐cisplatin (LLC) drug, the number of platinum‐DNA adducts increased significantly in LLC‐treated insensitive cells. Moreover, the cisplatin content in LDs increased upon LLC treatment. Furthermore, lipid profiles of 22 carcinoma cells with differential cisplatin sensitivity (9 sensitive vs 13 insensitive) were acquired. These profiles revealed low storage lipid levels in insensitive cells. This result recommends that LD lipidome might be a common pathway in multiple cancers for platinum sensitivity in EOC. Finally, LLC suppressed both cisplatin‐insensitive human carcinoma cell training and testing sets. Thus, LDLR‐platinum insensitivity can be due to a defective Lands cycle that hinders LPC production in LDs. Using lipidome assessment with the newly formulated LLC can be a promising cancer chemotherapy method.

## 1 INTRODUCTION

Platinum‐based chemotherapy is the primary modality used for treating patients with solid tumours. Platinum is often considered a first‐line chemotherapy drug.^1^, ^2^, ^3^ Moreover, platinum has been combined with various chemoagents and used for treating cancers for which effective drugs are unavailable to date.^3^ However, the clinical utility of platinum is limited because of the resistance of certain types of cancers to platinum and normal tissue toxicities, which are determined by the level of platinum accumulation in tissues.^1^ Among the various reported mechanisms, the most acceptable hypothesis in this field was the expression of ATP‐binding cassette (ABC) gene family in cancer cells.^4^, ^5^ The expression of the ABC gene family can pump the intracellular chemotherapy drug out of the cells to avoid the cytotoxic effect.^6^ The use of an ABC gene blocker was tested for treating multiple drug resistance; however, the results were controversial. For instance, in a study, ABC blockers were used in multiple trials.^7^ However, no evidence suggests ABC blockers to be an effective second‐line chemotherapy drug.

Lipids, essential biological building blocks, can act as bioactive molecules, such as they can be constituents of cellular membranes or as a supplier of a sufficient amount of energy for the fast‐growing nature of cancer cells.^8^ One of the most important metabolic markers of cancer cells is the deregulation of lipid metabolism.^9^ Recent studies have shown that lipid metabolism plays crucial roles for providing energy, macromolecules for membrane synthesis and lipid signals during cancer progression.^10^ Moreover, lipid droplet (LD) accumulation in cancer cells is a pivotal adaptive response to deleterious conditions.^11^

Lipid importation from circulation, other than endogenous lipid synthesis, affects cancer progression has been attracting tremendous attention in the field of cancer metabolism.^12^ In a bioinformatics study, Li et al^13^ found that low‐density lipoprotein receptor (LDLR) expression is an important biomarker for renal cell carcinoma. Cholesterol importation occurs in pancreatic cancer potentially through LDLR expression in cancer cells.^14^ Moreover, cholesterol importation^15^ to steroidogenic enzymes except endogenous cholesterologenesis through LDLR and SR‐D1 scavenger receptor is the prognostic biomarker of gastric cancer (GCa).^16^ Targeting the steroidogenic enzyme cytochrome CYP450 19A1 (aromatase) with exemestane can be effective for patients with GCa.^17^

Study shows that differential levels of LDLR expression in EOC cells determine the platinum sensitivity in an LDLR‐dependent manner.^10^ LDLR expression reprogrammes cellular transcriptome associated with lipid metabolism (Lands cycle in LD) to be the mechanism underlying cisplatin sensitivity. Moreover, lysophosphatidylcholine (LPC) acyltransferase 1/2 (LPCAT1/2), a Lands cycle enzyme, has been recognized as a key chemoresistance molecule in multiple cancers.^18^, ^19^

Abundant evidence has indicated that intracellular lipid resources, either endogenous or exogenous, are the key biochemical event indicating chemotherapy responsiveness of multiple cancers. In the present study, we explored LDLR‐mediated lipidome alteration for platinum therapy sensitivity by using lipidomics and bioinformatics approaches for illustrating cellular lipidome and testing the hypothesis that targeting lipidome, instead of gene expressions, is a useful therapeutic strategy.

## 2 MATERIALS AND METHODS

### 2.1 Reagents, cell culture and lentiviral‐based gene delivery

Cells were maintained in various culture media depending on the culture requirements with 10% FCS (foetal calf serum; Invitrogen), 1% L‐glutamine and 1% penicillin‐streptomycin, as described previously.^15^ An HEK293T cell line—HTB52—and EOC cell lines—MDAH‐2774; SKOV3, HTB‐77; OVCAR3, HTB‐161; ES2, CRL‐1978; TOV‐112D, CRL‐11731; and TOV‐21G, CRL‐11730—were purchased from ATCC. Head and neck squamous cell carcinoma cell lines—OECM1, FaDu and SAS—were kindly provided by Prof. Kou‐Juey Wu of China Medical University (CMU); PanCa cell lines—CFPAC‐1, HPAF‐II, ASPC‐1 and BxPC‐3—by Prof. Wen‐Hwa Lee of CMU; HCC cell lines—Tong, HCC36, Huh7 and HepG2—by Dr YS Jou of Academia Sinica; a renal carcinoma (RCC) cell line—769‐p—by Prof. Chawnshang Chang of the University of Rochester, NY, USA; and the CC (Cholangiocarcinoma) cell line—H1, RBE and SSP25—by Prof. Chiung‐Kwei Huang of Brown University (RI, USA). GCa cell lines—AGS, MKU‐1 and SC‐M1—were purchased from the Food Industry Research and Development Institute, Taiwan (BCRC purchase number: 60210).

The following antibodies were used: anti‐LDLR (sc‐373830, immunoblot: Santa Cruz), anti‐perilipin‐2 (NB110‐40877, Novus Biological Ltd.), anti‐DNA adduct [CP9/19] (Abcam, 1:1000 dilution), anti‐actin (sc‐47778, Santa Cruz) and anti‐tubulin (ab‐6046, Abcam) antibodies. Moreover, cisplatin (P4394, Sigma‐Aldrich), 1,2‐dioleoyl‐3‐trimethylammonium‐propane (DOTAP; 890890P, Avanti) and LPC (855675P, Avanti) were used.

### 2.2 Lipid profiling for lipidome analysis

After the cells (1500 cells/µL × 300 µL) were washed with Ca^2+^‐ or Mg^2+^‐free PBS, the lysates were subjected to lipid profiling executed by Lipotype GmbH.^20^, ^21^, ^22^ Lipidomes were prepared from at least three replicates of each sample for all the experiments by using the subsequently described procedures.

#### 2.2.1 Nomenclature

The following lipid names and abbreviations were used: Cer, ceramide; Chol, cholesterol; DAG, diacylglycerol; HexCer, glucosyl/galactosylceramide; SE, sterol ester; SM, sphingomyelin; SL, sphingolipid; TAG, triacylglycerol; PA, phosphatidic acid; PC, phosphatidylcholine; PE, phosphatidylethanolamine; PG, phosphatidylglycerol; PI, phosphatidylinositol; and PS, phosphatidylserine. Their lysospecies are lyso‐PA (LPA), lyso‐PC (LPC), lyso‐PE (LPE), lyso‐PI (LPI) and lyso‐PS (LPS), and their ether derivatives are PC‐O, PE‐O, LPC‐O and LPE‐O. Lipid species were annotated based on their molecular composition as follows: [lipid class]‐[sum of carbon atoms in the fatty acids]:[sum of double bonds in the fatty acids];[sum of hydroxyl groups in the long‐chain base and the fatty acid moiety] (eg SM‐32:2;1). Individual fatty acid compositions following the same rules were provided in parentheses where available (eg 18:1;0‐24:2;0). For the categorization of lipid species, the major cellular location of lipid moiety denoted the categorization of storage (STO), membrane (mem) or lysosomal (LYS). For example, TAG and DAG are membrane lipids; cholesterol esters are storage lipids; lysophospholipids are lysosomal lipids.

#### 2.2.2 Lipid extraction for mass spectrometry lipidomics

Lipids were extracted using a two‐step chloroform‐methanol procedure. Samples were spiked with an internal lipid standard mixture containing cardiolipin (CL), 16:1/15:0/15:0/15:0; Cer, 18:1;2/17:0; DAG, 17:0/17:0; HexCer, 18:1;2/12:0; LPA, 17:0; LPC, 12:0; LPE, 17:1; LPG, 17:1; LPI, 17:1; LPS, 17:1; PA, 17:0/17:0; PC, 17:0/17:0; PE, 17:0/17:0; PG, 17:0/17:0; PI, 16:0/16:0; PS, 17:0/17:0; cholesterol ester (CE), 20:0; SM, 18:1;2/12:0;0; TAG, 17:0/17:0/17:0. After extraction, the organic phase was transferred to an infusion plate and dried in a speed vacuum concentrator. Each first‐step dry extract was resuspended a 1:2:4 (v/v/v) chloroform‐methanol‐propanol in 7.5 mmol/L ammonium acetate, and each second‐step dry extract was resuspended in a 0.003:5:1 (v/v/v) methylamine‐chloroform‐methanol in 33% ethanol. All liquid handling steps were conducted using the Hamilton Robotics STARlet robotic platform with the Anti‐Droplet Control feature for organic solvent pipetting.

#### 2.2.3 Mass spectrometry data acquisition

Samples were analysed by direct infusion on a Q Exactive Mass Spectrometer (Thermo Scientific) equipped with the ion source TriVersa NanoMate (Advion Biosciences). Samples were analysed in both the positive and negative ion modes at a resolution of 280 000 at an m/z of 200 for mass spectrometry (MS) and 17 500 for tandem MS (MS/MS) experiments in a single acquisition. MS/MS was triggered by an inclusion list comprising the corresponding MS mass ranges scanned in 1‐Da increments. Both MS and MS/MS data were combined to monitor CE, DAG and TAG ions as ammonium adducts; PC and PC‐O as acetate adducts; and CL, PA, PE, PE‐O, PG, PI and PS as deprotonated anions. Only MS was used to monitor LPA, LPE, LPE‐O, LPI and LPS as deprotonated anions. Moreover, Cer, HexCer, SM, LPC and LPC‐O were monitored as acetate adducts and Chol as an ammonium adduct of an acetylated derivative.^23^

#### 2.2.4 Data analysis and post‐processing

Lipid identification was performed on unprocessed mass spectra by using LipotypeXplorer (2). For the MS‐only mode, lipid identification was based on the molecular masses of the intact molecules. The MS/MS mode involved the collision‐induced fragmentation of lipid molecules. Moreover, lipid identification was based on both intact and fragmented masses. Before normalization and further statistical analysis, the lipid identifications were filtered, based on mass accuracy, occupation threshold, noise and background characteristics. The lists of the identified lipids and their intensities were stored in a database optimized for the particular structure inherent to lipidomic data sets. Lipid class‐specific internal standards’ intensity was used for lipid quantification.^24^ The identified lipid molecules were quantified using normalization to a lipid class‐specific internal standard. The amount of individual lipid molecules (species of subspecies) in p moles of a given lipid class was summed to yield the total amount of the lipid class. The lipid class amounts may be normalized to the total lipid value by yielding the mol.% for the total lipid amount.

### 2.3 Lipid data processing

The lipid profiling data of each sample were scale‐normalized by the total amount of lipid. The lipids with at least a twofold change between the LDLR knockdown cells and control cells were identified as lipids significantly regulated by LDLR. Then, Fisher's exact test was conducted to test the enrichment of the significantly regulated lipids for each lipid class, such as PC, PE and LPC.

### 2.4 Lentiviral‐based gene delivery

LDLR knockdown cells or overexpression clone cells were engineered by the stable transfection of human LDLR cDNA (pLenti‐C‐mGFP‐LDLR, RC200006L2; OriGENE) or pLKO.1‐shLDLR (targeting sequence: 5′‐GGG CGA CAG ATG CGA AAG AAA)^10^ and then selected after exposure to puromycin (10 μmol/L) for a month.^25^, ^26^, ^27^ The pLKO‐shLuciferase plasmids were obtained from the National RNAi Core Facility Platform (Institute of Molecular Biology or Genome Research Center, Academia Sinica, supported by the National Core Facility Program for Biotechnology; grant number: NSC107‐2319‐B‐001‐002). The lentiviral production and infection procedures used in this study followed those reported previously.^25^ In brief, psPAX2 (packaging plasmid) and pMD2G (envelope plasmid) (Addgene) were cotransfected into the HEK293T cells. Then, the virus‐containing media were harvested to infect the HCC cells. The GFP + cell populations, as determined by the flow cytometry analysis (BD LSR II Flow Cytometry), were used to test the infection efficiencies.

### 2.5 The Cancer Genome Atlas database DriverDB (version 2) and Kaplan‐Meier plotter meta‐analysis for cancer survival analysis and algorithm for hazard ratio scoring

DriverDB,^28^, ^29^ a database that incorporates >9500 cancer‐related RNA‐Seq data sets and >7000 exome‐seq data sets from The Cancer Genome Atlas (TCGA), was used in this study. DriverDBv3^30^ comprises 420 primary tumour data and 37 adjacent normal tissue data (including 34 normal‐tumour pair data) in the EOC data set in TCGA. For conducting the survival analysis of TCGA data, Kaplan‐Meier (KM) survival curves were drawn. Moreover, a log‐rank test was performed to assess the differences between the patient groups stratified by the median of gene expression. A *P* value of <.05 was considered statistically significant.

In the web‐based KM plotter platform, the following previously established formula was used^15^ to evaluate the hazard ratios (HRs) of the pathways (cluster of genes) with respect to patient survival:

(1) $$\text{HR score}=\left(\text{Avg. of HR of gene sets}\right)=\frac{\sum \left(\text{HRn}\;-\;\text{1}\right)\times \left(-\log_{10}\left(P-\text{value}\right)\right)}{n}$$

To evaluate the influence of each gene, the absolute HR for that gene minus 1 was calculated. To adjust for the gene effects, the HR of each gene was multiplied by a negative log_10_ *p* for balancing the importance of the genes. The summed score was then divided by the number of genes and multiplied by 100 to obtain the HR or average HR of all the genes.

### 2.6 Doubling time and 50% inhibition concentration measurement

For the cell viability assay, cells were seeded in 96‐well plates (5 × 10^3 ^cells/well) and incubated overnight for attachment. These cells were then treated with the indicated drug doses in normal media for 48 hours. After the treatments, the media were replaced with MTT assay (0.5 mg/mL) at 37°C for at least 1 hour. After the removal of excess WST‐1 (Sigma‐Aldrich), the colorimetric absorbance of the cells at 490 nm was recorded. The measured values of 50% inhibition concentration (IC_50_)^31^ for each drug were determined using the CalcuSyn software^32^ (BioSoft).

The calculation of the doubling time of cell lines in this study was the same as the procedure described on http://www.doubling‐time.com/compute.php and calculated using the following equation:

(2) $$\text{Doubling time}\;=\;\frac{\text{duration}*\log\left(2\right)}{\log\left(\text{final concentration}\right)-\log\left(\text{initial concentration}\right)}$$

In brief, cells were seeded at 2 × 10^4^ cells/6‐well dishes, and the cells were collected after 24, 48, 72, 96 and 120 hours of seeding. Then, the cells were placed on a cell counting chamber slide, and the cell concentration was recorded.

### 2.7 LPC‐liposome‐cisplatin preparation and characterization

The liposome was prepared using a thin‐layer hydration,^33^ followed by the application of the membrane protrusion method^34^ with some modifications. First, we hydrated a 1:1 molar mixture of DOTAP [molecular weight (MW) = 698.5 g]‐cholesterol or LPC (MW = 495.63 g)‐cholesterol (MW = 386.6 g) by using double distilled water (Milli‐Q Plus, Millipore). The mixture was incubated at 65°C for 1 hour and then sonicated in a 65°C water bath for 30 minutes. The mixture was then subjected to membrane protrusion (Mini‐Extruder, Avanti Polar Lipid, Ltd.) by using a 200‐nm pore size membrane (Avanti Polar Lipid, Ltd.) that is extruded 20 times to form preliposomes. The preliposomes were then subjected to protrusion with a 100‐nm pore size membrane that is extruded another 15 times. The size and size distribution of the liposome were then determined by photon correlation spectroscopy (Zetasizer Nano ZS90, Malvern Instruments Ltd.). Liposomes (10 µL) were dispersed with 500 µL of purified water in a low‐volume disposable sizing cuvette. The particle size and size distribution were measured in terms of the ZAve and polydispersity index, respectively.

### 2.8 Experimental animal and xenograft implantation tumour model

Athymic nude female mice aged 6‐8 weeks old (*Foxn1^nu^*) were purchased from the National Laboratory Animal Center (NLAC), Taiwan. Subcutaneous implantation of 1 × 10^6^ cells/100 μL PBS and Matrigel (1:1) in both flanks was performed on each mouse. The mice were then randomly divided into experimental groups as the tumours grew to 200 mm^3^, and the size of each tumour was measured twice/week. The mice were treated with either DLC or LLC (intratumoral injection, every other day for 3 weeks). The mice were then killed, and the tumours were harvested. All the animal studies were performed under the supervision, guidelines and approval of the China Medical University Animal Care and Use Committee (#CMOIACUC‐2018‐089). The formulation to calculate Tumor Suppression Index (TSI) follows the equation below:

(3) $$\text{TSI}=\frac{\text{LLC tumor size}\;\left(\text{end point}-\text{initial}\right)}{\text{DLC tumor size}\;\left(\text{end point}-\text{initial}\right)}\times \%$$

### 2.9 Cisplatin‐DNA adduct measurement with flow cytometry

Platinum‐DNA adduct measurement was described previously.^35^ In brief, the cells were subjected to various treatments for 24 hours to form cisplatin‐DNA adducts. Then, these cells were harvested using PBS containing 0.1% Triton X‐100 and fixed with 70% ethanol. Subsequently, the cells were washed with PBS and stained with an anti‐cisplatin‐modified DNA [CP9/19] antibody (Abcam, 1:1000 dilution) overnight at 4°C. The cells were then stained using a goat anti‐rat FITC‐conjugated secondary antibody for 2 hours. The signals of the cisplatin‐DNA adducts were detected through flow cytometry (BD Biosciences). The data were analysed using FCS Express (version 3.0; De Novo Software).

### 2.10 LD isolation

The LD isolation was conducted using a published protocol.^36^, ^37^ In summary, the cells were cultured with 20% FCS in a 150‐mL flask. Approximately 1‐2.5 × 10^7^ cells were harvested for LD isolation. Before collection, the cells were washed with ice‐cold PBS in 15‐mL tube by centrifugation for 10 minutes at 1000 *g*, 4°C. Remove the supernatant by aspiration with a pipette, and gently and thoroughly resuspend the cell in ice‐cold hypotonic lysis medium (HLM; 20 mmol/L Tris‐HCl, pH 7.4; 10 mmol/L sodium fluoride; 1 mmol/L EDTA; protease inhibitor). The mixture was allowed to sit on an ice‐cold HLM for 10 minutes. The sediments were homogenized with a Bio‐Gen PRO200 Homogenizer. The homogenized lysate was then centrifuged (1000 *g*, 10 minutes, 4°C), and the supernatant was collected. Add 1/3 volume of ice‐cold HLM medium containing 60% sucrose and mix by gentle pipetting. Then, cell lysate subjected into the bottom of a 13.2‐ml ultracentrifuge tube for an SW 41 Ti rotor, gently layer 5 mL ice‐cold HLM containing 5% sucrose and gently add 5‐6.5 mL ice‐cold HLM over the sucrose layers to fill the tube. The stratification was then centrifuged (28 000 *g*, 30 minutes, 4°C), and a floating white layer formed (LD) was transferred to new plastic tube by using a Pasteur pipette. The liquid underneath the white layer also collected as the non‐LD counterpart.

### 2.11 Quantitation of cisplatin uptake by cells

The platinum quantitation method for cells was modified from a previous study.^38^ The platinum levels in cells were detected and measured using inductively coupled plasma (ICP)‐MS. The cells were treated with 0.5 mL each of 65% nitric acid and 30% H_2_O_2_ for digestion, followed by heating up to 80°C for 1 hour. After digestion, 0.5 mL of 25% ammonia water was added to the cell samples to neutralize the excess nitric acid. The samples were then diluted using ultrapure water of up to 4 mL. Moreover, the platinum level measurement was conducted using an ICP‐MS system (Agilent 7900 ICP‐MS, Agilent 7500a, Agilent Technologies) in a certified laboratory (Super Micro Mass Research and Technology Center, Cheng Shiu University).

### 2.12 Statistical analysis

Student's *t* and chi‐square tests were conducted to identify the significant differences between groups and categorical variables. A *P* of <.05 was considered significant. All data were presented as means ± standard errors of the means.

## 3 RESULTS

### 3.1 LDLR‐mediated LD lipidome alteration for cisplatin sensitivity

**Figure 1:**
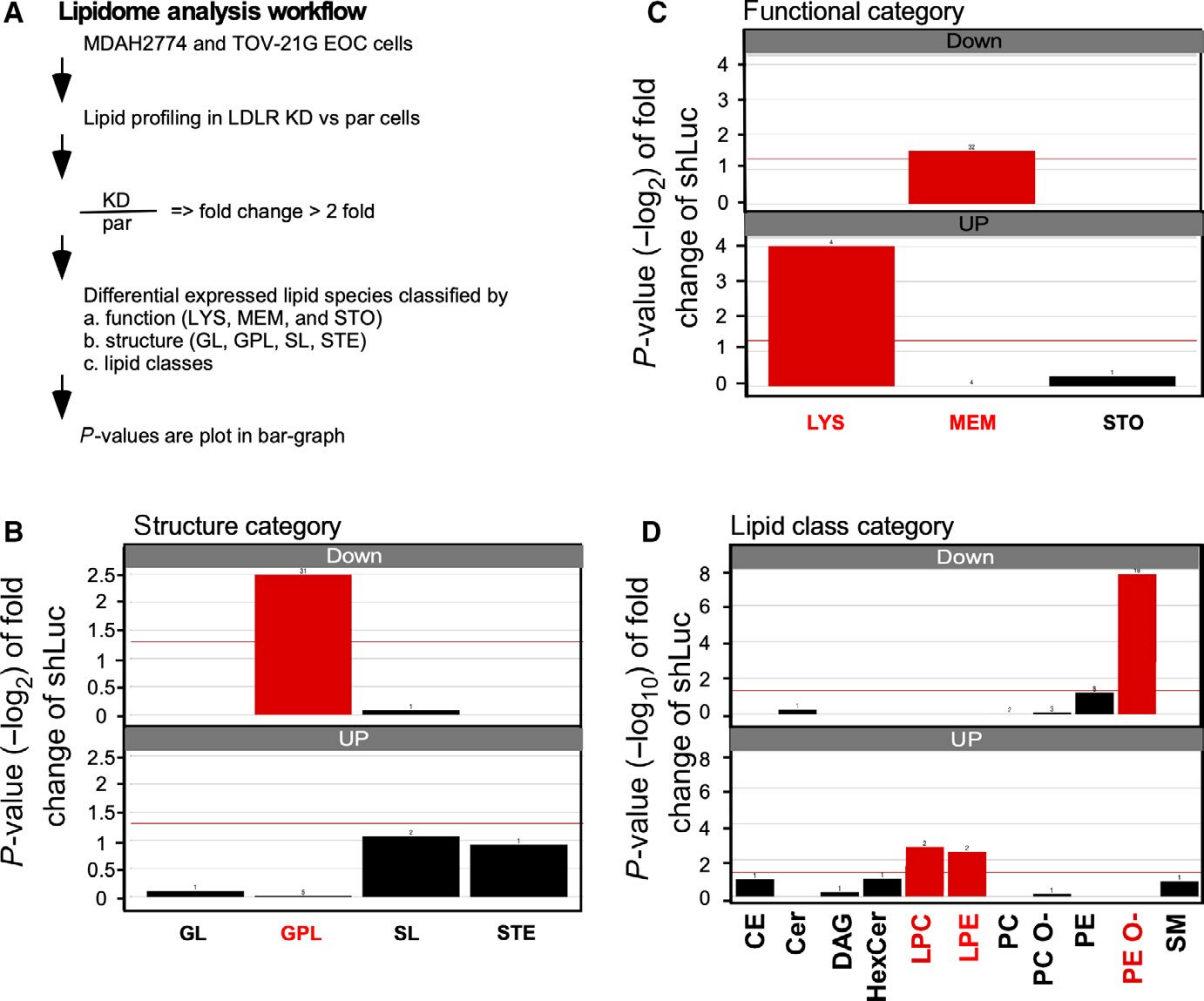
Lipidome analysis conducted on cells obtained from MDAH‐2774 and TOV‐21G LDLR knockdown cells. A, Experimental design and data analysis strategy for the lipidomic analysis. MDAH‐2774 and TOV‐21G parental (par) cells vs low‐density lipoprotein receptor (LDLR) knockdown (KD) cells were harvested, and lipid profiling experiments were conducted. The lipid species alteration (lipid expressions of KD cells divided by that of par cells) larger than ±2 folds were subjected to different category enrichment methods, such as functional (LYS: lysosomal; MEM: membrane; and STO: storage lipids), structural (GL: glycerolipid; GPL: glycophospholipid; SL: saccharolipid; and STE: steroidal) and lipid classes (total of 30 species lipids). B, Structure category enrichment result of the lipidomic analysis. The down‐regulated lipids were significantly enriched in the GPL lipids (red). C, Function category enrichment result of the lipidomic analysis. The down‐regulated lipids were significantly enriched in MEM, and the up‐regulated lipids were significantly enriched in LYS lipids (red). D, Lipid class category enrichment result of the lipidomic analysis. The down‐regulated lipids were significantly enriched in PE‐O, and the up‐regulated lipids were significantly enriched in LPC and LPE (red). Abbreviations: CE, cholesterol ester; Cer, ceramide; DAG, diacylglycerol; HexCer, hexosylceramide; LPC, lysophosphatidylcholine; LPE, lysophosphatidylethanolamine; PC, phosphatidylcholine; PC‐O, ether‐linked phosphatidylcholine; PE, phosphatidylethanolamine; PE‐O, ether‐linked phosphatidylethanolamine; SM, sphingomyelin

**Figure 2:**
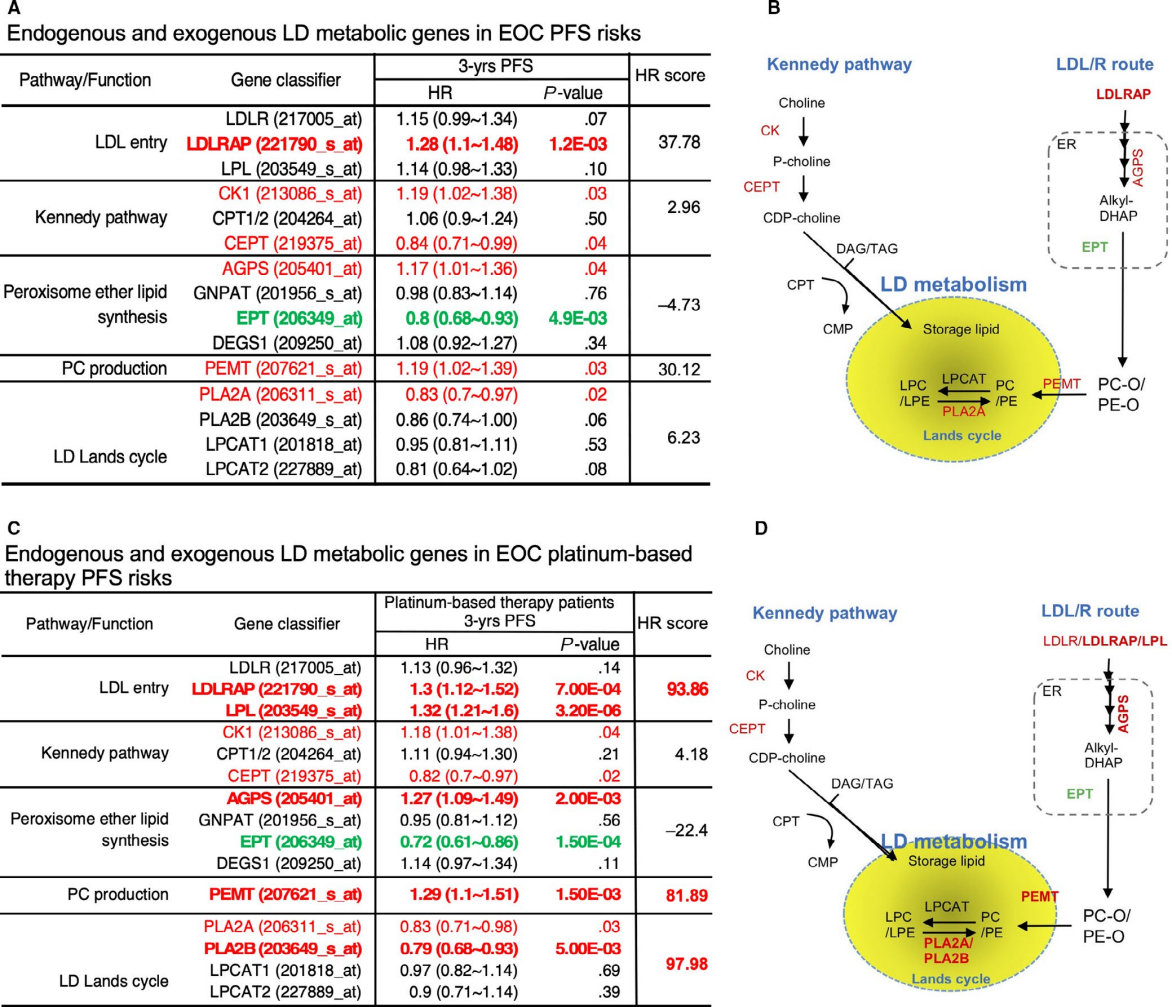
Hazard ratio (HR) analysis with genes related to lipid droplet (LD) lipid resources. A and C, Summary table that analyses genes, such as LDL entry, Kennedy pathway, peroxisome ether lipid synthesis, PC production and Lands cycle. A and C, summarize the HR scores of patients with EOC who did not receive and received platinum therapy, respectively. The LDL entry gene includes LDLR; LDLR‐associated protein (LDLRAP); LPL (lipoprotein lipase); and Kennedy pathways such as choline kinase (CK1), choline phosphotransferase (CPT) and choline/ethanolamine phosphotransferase (CEPT). Peroxisome ether lipid synthesis include alkylglycerone phosphate synthase (AGPS), glyceronephosphate O‐acyltransferase (GNPAT), CDP‐ethanolamine:DAG ethanolamine phosphotransferase (EPT) and delta 4‐desaturase sphingolipid 1 (DEGS1). Here, PC production is phosphatidylethanolamine *N*‐methyltransferase (PEMT), Lands cycle includes phospholipase 2A or 2B (PLA2A or PLA2B) and LPC acyltransferase 1/2 (LPCAT1/2). The 3‐year progression‐free survival (PFS) data, HR and *P* value were listed and subjected to the pathway HR score analysis (Equation 1). The cut‐off value of the HR score was set to 50 as significant impact to PFS (table on the right). B and D, Lipid resource of LD that includes neutral lipids from the Kennedy pathway (image on the left‐hand side) or phospholipids from the LDL/R route peroxisome (image on the right‐hand side). Red‐labelled genes indicate a significant HR score that facilitates PFS, and the green‐labelled genes related to HR inhibit PFS. The bold font indicates the gatekeeper genes that dominate PFS

As mentioned above, LDLR‐insensitized EOC cells respond to the cisplatin treatment. Thus, the cellular lipidome alteration was a topic of interest. To understand how LDLR expression affects the cellular lipidome alteration, LDLR knockdown experiments were conducted in MDAH‐2774 and TOV‐21G EOC cells with strong LDLR expression. The lipid profiles that varied by more than twofold were subjected to functional, lipid structure and class enrichment analyses (Figure 1A). In terms of the lipid structure category, the glycophospholipid expression was significantly down‐regulated (Figure 1B). For the lipid function category, the membranous lipids were up‐regulated, and lysosomal lipids were down‐regulated. A detailed lipid class enrichment revealed that PE‐Os were significantly up‐regulated and that LPCs were significantly down‐regulated. Because PE‐O can be converted into phosphatidylcholine (PC) via phosphatidylethanolamine *N*‐methyltransferase (PEMT) from peroxisome to LD,^39^, ^40^ the LDLR→PE‐O production may be contributing to LD anabolism. Moreover, by considering the lipid classes (LPC vs PE‐O) and the related functional and structural enrichment, we speculated that the LDLR‐lipidome alteration might affect LD genesis, which leads to platinum insensitivity. Therefore, we used a hypothesis‐driven web‐based survival analyser with an established algorithm^15^ to test genes and prognosis correlation. The KM plotter is used for associating gene expressions to 3‐year progression‐free survival (PFS) in EOC patients receiving platinum therapy (hereafter, platinum‐treated patients; Figure 2A) and who did not receive platinum therapy (hereafter, general patients with EOC, Figure 2C).

The genes involved in lipid metabolism along with organelles are illustrated in Figure 2B,D. The lipid metabolism also includes the neutral lipid resources from the Kennedy pathway (LD neutral lipid resources) and peroxisome ether‐linked phospholipid synthesis [eg PE‐O or PC‐O lipid resources from endoplasmic reticulum (ER) and peroxisome ^41^; Figure 2B,D]. The Kennedy pathway genes use choline kinase (phosphorylates choline), choline phosphotransferase (CPT, the enzyme used catalyses cytidine triphosphate (CTP) and choline phosphate to form CDP‐choline (cytidine diphosphate‐choline)) and choline/ethanolamine phosphotransferase (CEPT) to import neutral lipids (eg DAG and TAG) into LD.^42^, ^43^, ^44^ By contrast, the entry of lipids from the LDL/R route [eg LPL and LDLR‐associated protein (LDLRAP)] was consequently through alkyl glycerone phosphate synthase (AGPS) in ER,^45^ CDP‐ethanolamine:DAG ethanolamine phosphotransferase (EPT) and PEMT from peroxisome to import PE‐O or PC‐O into LD.^46^ Finally, the Lands cycle enzymes, such as LPC acyltransferase (LPCAT) or phospholipase 2A or 2B (PLA2A or PLA2B), conduct the PE‐PC and LPE‐LPC conversions in the LD.^47^ The LPC‐LPC conversion product could then consume neutral lipids, thus accomplishing LD metabolism.

We found that high LDLR uptake genes (eg LDLRAP and LPL) were associated with a poor PFS (Figure 2A,C). However, by calculating the effect of multiple genes on PFS, we found that LDLR uptake genes had a more severe influence on platinum therapy patients (evidenced by the differences in HRs calculated using Equation 1) than that on general patients with EOC. These findings are consistent with the laboratory observations pertaining to the LDL/R route‐unsensitized chemotherapy. As aforementioned, the conversion from PC to LPC and vice versa is crucial in LD metabolism. Therefore, we examined HRs of enzyme and gene clusters that are responsible for the endogenous lipid resources of LDs (ie the Kennedy pathway) (Figure 2A,B) and obtained a low HR. However, the Lands cycle enzymes, that is, PLA2A and PLA2B, are major risk factors in the PFS of platinum‐treated patients (Figure 2C,D) and reflected a high HR in our examination. Although all the HRs of gene clusters were analysed using the taxol‐based therapy and PFS data of patients with EOC, low HRs were observed (data not shown), thus indicating that the use of the LDL/R route and a Lands cycle enzyme is a crucial lipid metabolic pathway for platinum therapy prognosis.

In summary, HR discrepancies between platinum chemotherapy and other chemotherapies suggested the unique function of the LDL/R route in the LD lipidome homeostasis remodelling for platinum sensitivity. Moreover, the data consistency between lipidome and bioinformatics studies suggested the potential of using LD lipidome targeting for platinum therapy.

### 3.2 Targeting LPC for manufacturing an efficacy‐boosting cisplatin‐liposome drug

**Figure 3:**
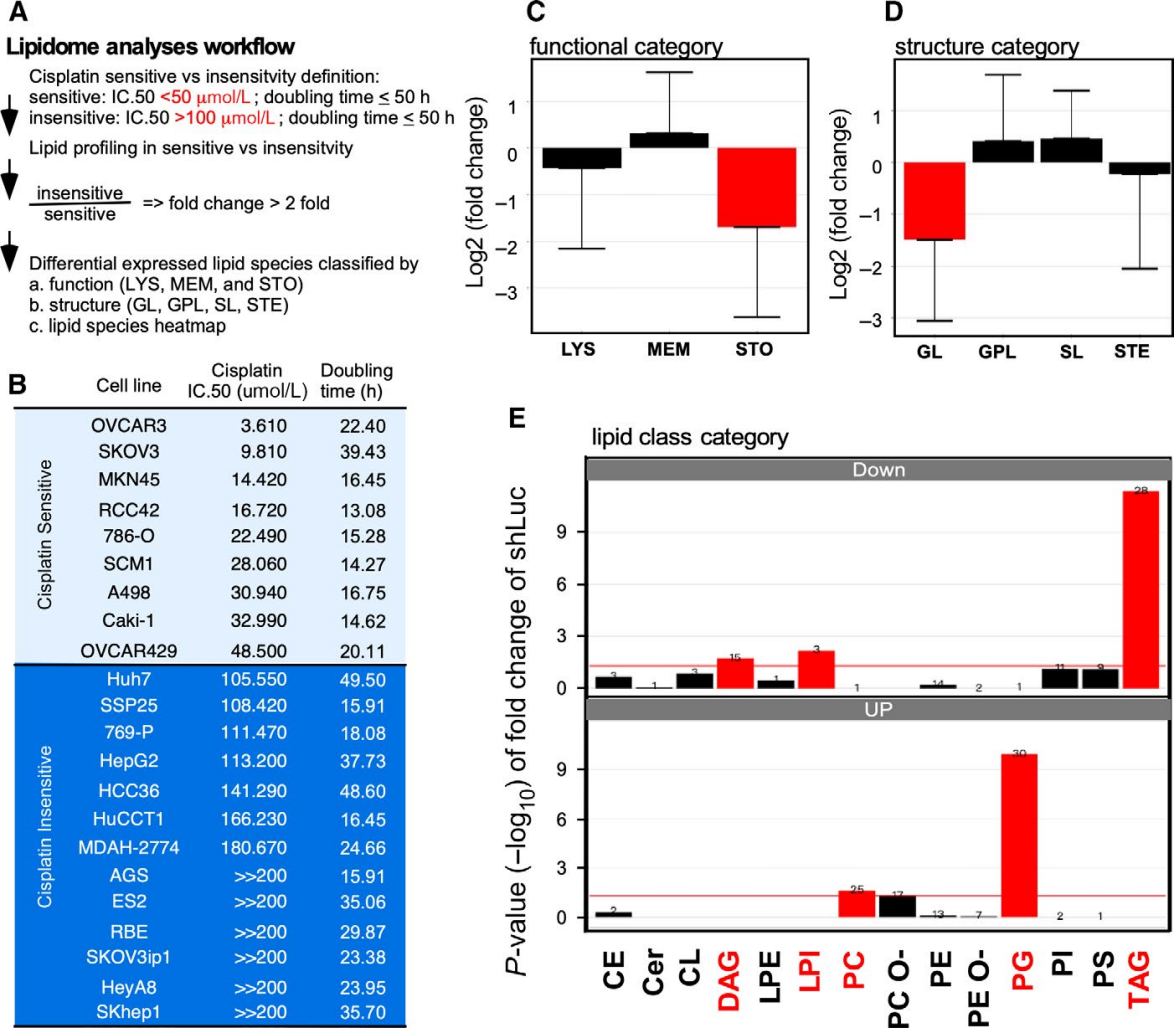
Lipidome analysis conducted on cisplatin‐sensitive and cisplatin‐insensitive cell lines. A, Experimental design and data analysis strategy for the lipidomic analysis. The cisplatin‐sensitive cells (nine cells) vs cisplatin‐insensitive cells (13 cells) were selected from 64 human carcinoma cells whose cisplatin cytotoxic was IC_50_. The doubling time for the basal cell growth rate and lipid profile were determined. The cisplatin sensitivity was defined to be IC_50_ < 50 µmol/L, where cisplatin insensitivity was defined to IC_50_ > 100 µmol/L. Moreover, a doubling time of >50 h was excluded. The lipid species alteration (lipid expressions of sensitive cells divided by that of insensitive cells) higher than ±2 folds were subjected to different categories’ enrichment, such as functional, structural or lipid classes. B, List of cell lines, including cisplatin‐sensitive and cisplatin‐insensitive cells. The light‐blue background represents the cisplatin‐sensitive cells, and the dark‐blue background represents the cisplatin‐insensitive cells. C, Function category enrichment result of the lipidomic analysis. The down‐regulated lipids were significantly enriched in the storage lipids (STO, red). D, Structure category enrichment result of the lipidomic analysis. The down‐regulated lipids were significantly enriched in the glycerolipids (GL, red). E, Lipid class category enrichment result of the lipidomic analysis. The down‐regulated lipids were significantly enriched in DAG, LPI and TAG, and the up‐regulated lipids were significantly enriched in PC and PG (red). Abbreviations: CE, cholesterol ester; Cer, ceramide; CL, cardiolipin; DAG, diacylglycerol; LPE, lysophosphatidylethanolamine; LPI, lysophosphatidylinositol; PC, phosphatidylcholine; PC‐O, ether‐linked phosphatidylcholine; PE, phosphatidylethanolamine; PE‐O, ether‐linked phosphatidylethanolamine; PG, phosphatidylglycerol; PI, phosphatidylinositol; PS, phosphatidylserine; TAG, Triacylglycerol

Notably, the discovery of the LDLR → LD lipidome → platinum therapy efficacy axis for targeting lipidome (eg LPC) could be of great significance in therapeutics. We conducted lipid profiling, defined as cisplatin cytotoxic at IC_50_, for testing this assumption and measured the doubling time for multiple cancer cells obtained from various origins (http://120.110.158.132:8787/trans_omics//lipidome_analysis.html). Moreover, a lipidomic analysis was conducted by segregating the cells into two groups: cisplatin‐sensitive and cisplatin‐insensitive cells. These cell groups were compared (cut‐off: IC_50_ < 50 µmol/L for cisplatin‐sensitive cells and IC_50_ > 100 µmol/L for cisplatin‐insensitive cells). A doubling time of <50 hours was selected (supposedly cisplatin kills fast‐growing cells, Figure 3A,B). The lipidome analysis that was conducted to compare cisplatin‐sensitive and cisplatin‐insensitive cells revealed that the storage lipid (Figure 3C) and glycerolipid (Figure 3D) levels were significantly lower in the sensitive cells than in the insensitive cells. A lipid class analysis exhibited an expression preference for sensitive and insensitive cells (Figure 3E). DAG, TAG and LPI were lower expressed, whereas PC and PG were higher expressed in sensitive cells compared to the insensitive ones (Figure 3E). These results indicate neutral lipid accumulation, which is related to LD abundance, in insensitive cells. Because LPC facilitates the dissolution of neutral lipids in LD,^47^ we considered using LPC for improving cisplatin efficacy.

**Figure 4:**
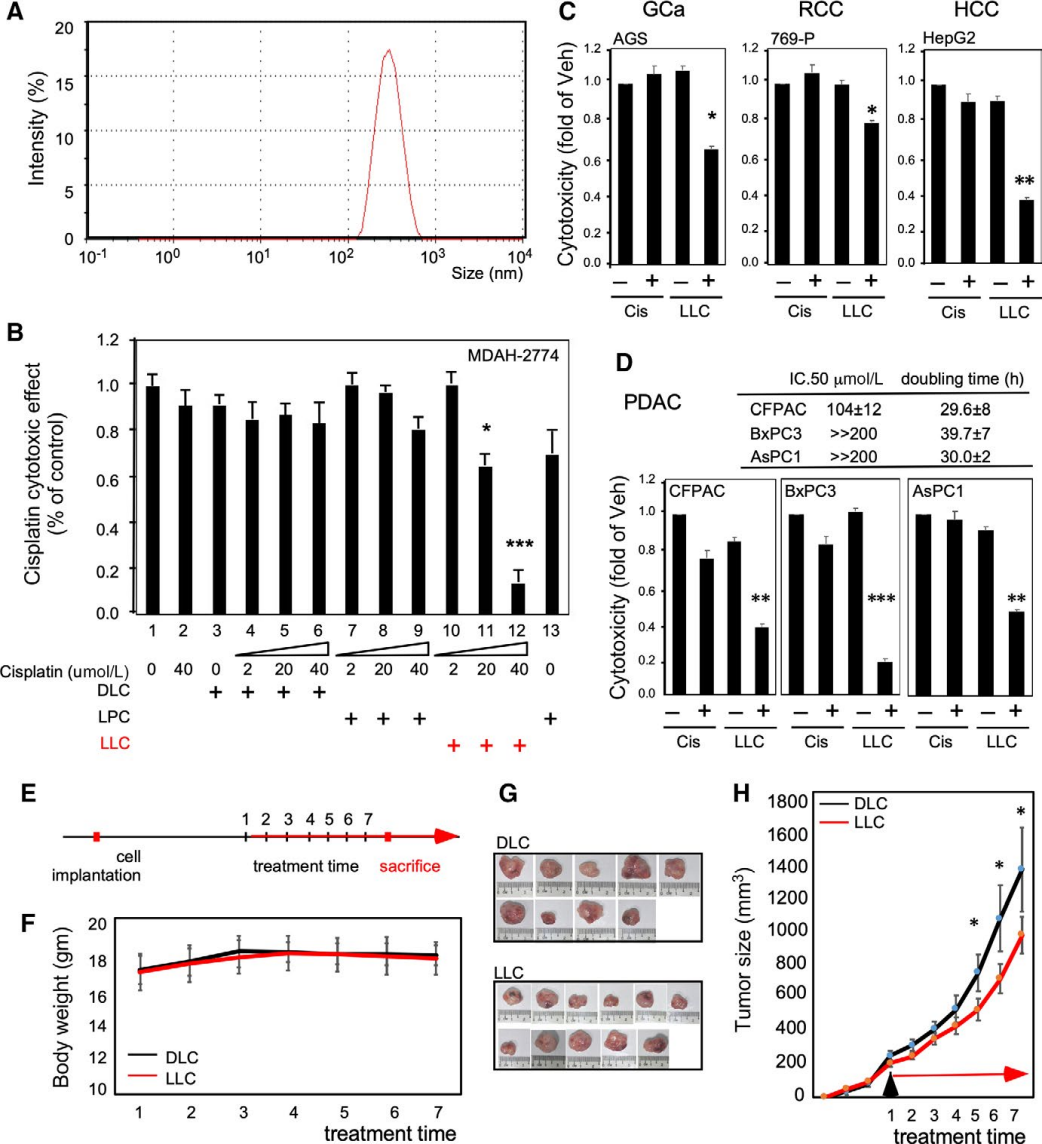
LLC production and effectiveness for cisplatin‐insensitive cells. A, Successful production of homogenous nanoscale liposome‐cisplatin drug by using LPC as a material (LPC‐liposome‐cisplatin, LLC). The Zetasizer presented a single‐peaked particle at approximately 200‐400 nm. B, Characterization of LLC by using only cisplatin (40 µmol/L, lane 2), regular liposome (0, 2, 20 and 40 µmol/L; DOTAP‐liposome‐cisplatin, DLC; lanes 3‐6), LPC and cisplatin co‐treatment (2, 20 and 40 µmol/L; lanes 7‐9), LLC (2, 20 and 40 µmol/L; lanes 10‐12) and only LPC. Red indicates the effective cytotoxic efficacy obtained using LLC. C, LLC suppressed cisplatin‐insensitive cells. AGS (gastric cancer; GCa), 769‐P (RCC) and HepG2 (hepatocellular carcinoma; HCC) that are listed in Figure 3B were introduced to test the cytotoxic efficacy (48 h treatment) of cisplatin (Cis; 20 µmol/L) and LLC (20 µmol/L cisplatin). D, LLC suppressed cisplatin‐insensitive cells. The pancreatic ductal adenocarcinoma cell lines (PDAC; CFPAC, BxPC3 and AsPC2) with unknown lipid profiles were introduced to test the cytotoxic efficacy of LLC (20 µmol/L cisplatin). The table on the upper panel displays the cytotoxic IC_50_ and the doubling time of the cell lines. The lower panels present the cytotoxic efficacy of LLC. All the cytotoxic efficacy experiments were conducted with 48‐h treatments. E, Experimental procedure of therapeutic efficacy comparison of LLC/DLC prototypes using xenograft EOC pre‐clinical model. F, Body weight change of xenograft tumour mice of DLC and LLC groups. G, Images of DLC‐ and LLC‐treated xenograft tumours at the time of sacrifice. H, Tumour growth curve on the MDAH‐2774 xenografted mice. Here, *P < .05, **P < .01 and ***P < .001 based on the *t* test results from at least three reproducible experiments

Liposome has been applied in pharmaceutics for decades as a drug carrier^48^, ^49^ and is particularly effective for delivering poor water‐soluble or highly toxic small‐molecule compounds.^50^ The addition of drug efficacy‐enhancing lipids while manufacturing liposomes has been proposed in pharmaceutics.^51^ However, there have been no tests on whether the lipid composition can be customized by varying the relationship between drug sensitivity and lipidome species. To test whether LDL/R → LD‐LPC → cisplatin sensitivity axis can be targeted, we formulated LPC‐liposome‐cisplatin (LLC) to test its cytotoxic efficacy. As shown in Figure 4A, we successfully manufactured LLC for a nanoscale homogenous liposome particle. For comparing the conventional liposome to the LLC, we compared the cytotoxic efficacy of DOTAP‐liposome‐cisplatin (DLC) with that of LLC. As shown in Figure 4B, the 40 µmol/L cisplatin cannot suppress cell growth (bar 1 vs 2). Moreover, DLC (incorporating 0, 2, 20 and 40 µmol/L cisplatin; bars 3‐6) treatments and co‐treatments involving LPC and cisplatin (2, 20 and 40 µmol/L; bars 7‐9) cannot suppress cell growth. Notably, an excellent dose‐dependent cytotoxic efficacy was observed when LLC (incorporating 2, 20 and 40 µmol/L cisplatin; bars 10‐12) was used on the MDAH‐2774 cisplatin‐insensitive cells. Moreover, the LPC treatment exhibited a cytotoxic level comparable with that of the vehicle treatment (bar 1 vs 13). This result indicates that targeting LPC for determining cisplatin sensitivity is vivid for drug efficacy boosting. Therefore, we used LLC on other cisplatin‐insensitive cells (Figure 3B) and found that LLC is very effective for suppressing cell growth in AGS (GCa); 769‐P (renal carcinoma); HepG2 (hepatocellular carcinoma) (Figure 4C); and CFPAC, BxPC3 and AsPC1 (pancreatic ductal adenocarcinoma, Figure 4D) cancer cells. This result indicates that the method of targeting LPC by using LLC could be applied to various types of cancers.

In order to test whether the LLC prototype can be used as potential therapy in the pre‐clinical model, we tested tumour suppression efficacy to compare LLC with DLC in the xenograft tumour model using MDAH‐2774 cisplatin‐insensitive EOC cells. As shown in Figure 4E, we implanted MDAH‐2774 cells s.c. into the flanks of female nude mice, allowed tumour to grow to ~200 mm^3^ and then started to treat DLC or LLC. As we observed the systemic toxicity with measuring body weight, we did not find obvious body weight change during treatments (Figure 4F). After treatment term, we found the tumour was smaller in LLC‐treated than in DLC‐treated group (Figure 4G,H). The Tumor Suppression Index is 66.28%. These data implicated a potential of implementing LLC in the future clinical application.

**Figure 5:**
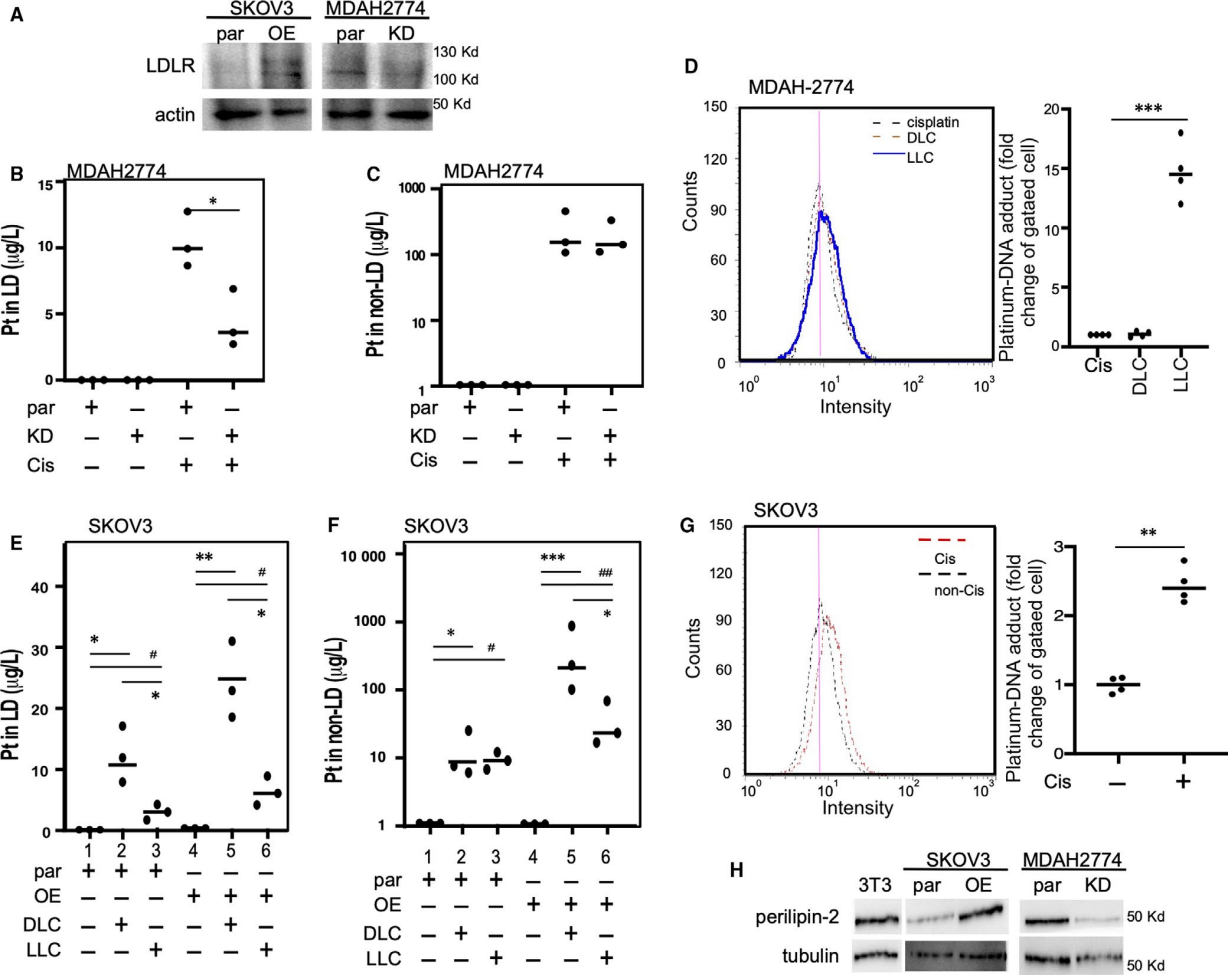
Manipulation of LDLR or LPC abundance could reprogramme LD metabolism and increase DNA adduct formation for enhancing the cisplatin cytotoxic effects. A, Establishment of LDLR overexpression (OE) in SKOV3 or KD cells in the MDAH‐2774 cell line and confirmation with immunoblot assay. Actin was measured as a loading control. B and C, LDLR KD in MDAAH‐2774 cells reduced the platinum amount (µg/L) in LD but sis did not alter the amount in the non‐LD counterpart. Platinum amount was measured by conducting ICP‐MS on LD extracts (B) or residuals (C) from par or KD cells that are with or without cisplatin (20 µmol/L) treatments. D, Platinum‐DNA adduct formation in MDAH‐2774 cells treated with cisplatin, DLC or LLC (all contained 20 µmol/L cisplatin). The platinum‐DNA adducts could be distinguished by the FITC‐conjugated antibodies and detected by flow cytometry. The histogram of flow cytometry presented on the left and quantitation presented on the right panel. E, LPC reduced platinum content in LD for both parental and LDLR OE SKOV3 cells. The platinum in LD (µg/L) was increased in the DLC‐treated parental cells (lane 1 vs 2), and lower LD content was observed while treating using LLC compared with the content obtained using the DLC (lane 2 vs 3). By contrast, a similar phenotype can be observed in the LDLR OE SKOV3 cells (lane 4 vs 5 or 5 vs 6). F, LPC reduced the platinum content in the non‐LD counterpart for both parental and LDLR OE SKOV3 cells. The platinum content in the non‐LD counterpart (µg/L) was increased in the DLC‐treated parental cells (lane 1 vs 2). During treatment, LLC exhibited a level comparable to the DLC (lane 2 vs 3). By contrast, LLC reduced the non‐LD platinum content in the LDLR OE SKOV3 cells (lane 5 vs 6). G, Platinum‐DNA adduct formation in SKOV3 cells treated with and without cisplatin (20 µmol/L cisplatin). The platinum‐DNA adducts can be distinguished by the FITC‐conjugated antibody and detected by flow cytometry. The histogram of flow cytometry is presented on the left, and quantitation is presented on the right panel. H: Perilipin‐2, which is an LD marker protein, was detected in OE and KD LDLR in SKOV3 and MDAH‐2774 cells, respectively. The expression of 3T3 cells served as a positive control of perilipin‐2 expression. The tubulin expression served as a loading control for LD. Here, * or ^#^ *P* < .05 and ** or ^##^ *P* < .01, based on the *t* test results from at least three reproducible experiments

As LPC can be targeted using LLC for cisplatin insensitivity, we examined whether this process was an LD‐related event. In this study, we used SKOV3 LDLR low‐expressed cisplatin‐sensitive cells and MDAH‐2774 LDLR high‐expressed cisplatin‐insensitive cells. Then, the LDLR expression levels were manipulated (Figure 5A) or LLC was used to measure the platinum amount in an LD and detect DNA adducts in the cells. As shown in Figure 5B,C, we compared the platinum amount in LDs isolated from MDAH‐2774 cells by using LDLR parental (par) cells and LDLR knockdown (KD) cells. The results revealed that LDLR knockdown could significantly reduce the platinum amount in the LDs (Figure 5B). By contrast, the amount of platinum in the non‐LD part is comparable for both parental and knockdown cells (Figure 5C). Based on the DNA adduct formation measurement (Figure 5D, left panel), the formation was higher in the MDAH‐2774 cells treated with LLC compared with the cells treated with cisplatin or DLC (Figure 5D, right panel).

DLC treatment exhibited a high effect (Figure 5E, lane 1 vs 2) but LLC treatment exhibited a slight effect on the platinum content in the LDs of SKOV3 parental cells (Figure 5E, lane 1 vs 3). A similar effect of DLC and LLC on the platinum content in the LDs can be observed in the LDLR OE SKOV3 cells (Figure 5E, lane 4 ~ 6). By contrast, both DLC and LLC treatments increased the platinum content in non‐LDs of SKOV3 parental cells (Figure 5F, lane 1 vs 2). By contrast, LLC exhibited less platinum content in the non‐LD part compared with the platinum content in LDLR OE SKOV3 cells when DLC treatment is used (Figure 5F, lane 5 vs 6). Based on the DNA adduct formation measurement (Figure 5G, left panel), DNA adduct was higher in the SKOV3 cells treated with cisplatin compared with the cells treated with vehicle (Figure 5D, right panel). The data in Figure 5E‐G are the reverse proof that LDLR‐‐>LPC‐‐>LD metabolism is the important mechanism for cisplatin sensitivity. Finally, we observed the expression of perilipin‐2 (an LD marker) in LDs from SKOV3 and MDAH‐2774 cells. We found that LDLR overexpression increased perilipin‐2 expressions in LD, but LDLR knockdown suppressed the expressions (Figure 5H). The data presented in Figure 5 suggest that altering LDLR, indeed, reprogrammed LD metabolism and could carry platinum. Therefore, platinum was eventually pumped out of the cancer cells. Moreover, altering LD metabolism by using LLC could improve the therapeutic efficacy.

## 4 DISCUSSION

### 4.1 Mechanism of LDLR‐LPC axis in platinum insensitivity

**Figure 6:**
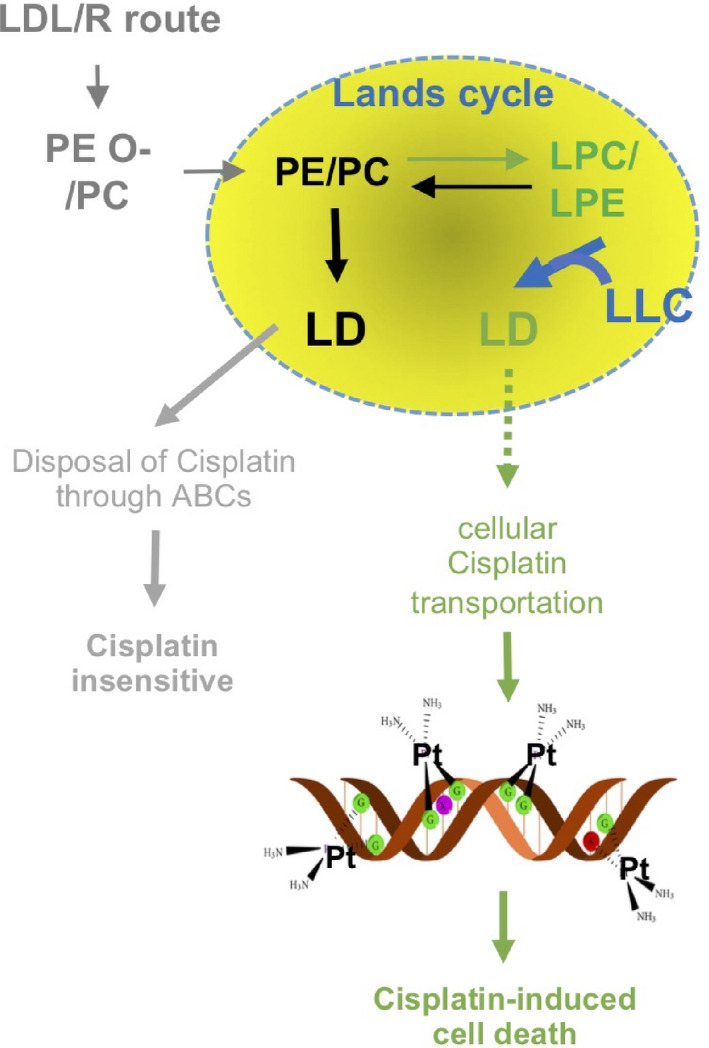
Mechanism of the LDL/R route‐mediated platinum insensitivity through Lands cycle lipids to increase LD anabolism and enhance platinum disposal through ABCs genes (grey). Targeting LPC to reprogramme LD metabolism could reduce the possibility of pumping platinum out of the cell. Thus, the possibility of platinum binding on DNA to form DNA adducts (green) was increased

We previously delineated the regulatory axis ‘LDLR→LPC→FAM83B→FGFRs’ for altering cisplatin sensitivity (not yet published manuscript). In the current study, we explored the LDLR‐related lipidome alteration in organelles and particularly focused on the LD function in terms of the platinum therapy efficacy. The major finding and significance of this study can be illustrated in Figure 6. LDLR expression can alter PE‐O and thus facilitate phospholipid entrance to LD (conversion by using PEMT, as shown in Figure 2). Moreover, the Lands cycle enzymes PLA2A and PLA2B or LPCAT1/2 (Figure 2) regulate LD metabolism. This regulation disposes cisplatin out of the cells through the ABC gene family, thus causing cisplatin insensitivity (Figure 6, in grey, on the left). However, targeting LPC by using LLC (Figure 6, in blue) could reprogramme LD metabolism. This facilitates cisplatin intracellular transportation and thus increases platinum‐DNA adduct formation in the cells. Finally, the increase in the platinum‐DNA adduct formation further elevates DNA mismatch repair and ROS stress, thus leading to cisplatin‐mediated cell death (Figure 6, in green).^52^

An issue observed based on the data presented in Figure 5F that the use of LLC could reduce the platinum amount in both LD fractions should be addressed. These data suggest that LD could partially explain the LDLR‐mediated cisplatin sensitivity. However, in Figure 5G, the platinum amount was also reduced in the non‐LD fraction. These data clearly suggest that LPC might also alter other signals for improving the cisplatin sensitivity. For instance, LPC is a signalling lipid that activates multiple signalling pathways that involve oxidative stress, inflammatory responses and Toll‐like receptors.^47^ Moreover, exogenous LPC induces proinflammatory effects; increases interleukin (IL)‐1β, IL‐6, interferon‐γ and tumour necrosis factor‐α (TNF‐α) secretion ^53^; and activates B cells and macrophages.^54^ LPC can enhance Foxp3 expression and suppress regulatory T cells possibly through G2A signalling.^55^

### 4.2 Targeting LD lipid remodelling for cancer therapy

LDs are intracellular lipid storage organelles that comprise a core of neutral lipids, such as DAG and TAG, and a surrounding monolayer of phospholipids, which is predominantly PC.^44^ The accumulation of LDs is a well‐recognized marker of cancer; however, the role of LDs in cancer is unclear to date. LD metabolism relies on the conversion of PC and LPC through the Lands cycle, which is dynamically balanced by LPCAT1/2/3 and phospholipase A.^44^, ^56^, ^57^ These lipid resources can be both endogenous and exogenous with endogenous lipids potentially contributed via the Kennedy pathway.^42^ Based on the bioinformatics analyses and in vitro validation conducted in this study, we observed significant influences of the LDL/R→ER/peroxisome → LD route, but not the Kennedy pathway, on the prognosis of EOC patients who received platinum therapies. For the first time, this study revealed the importance of exogenous lipid in LD metabolism. Some studies support this claim. For instance, a study of a colorectal cancer mouse model specified that LPCAT2 (LPC acyltransferase 2) contributed to LD accumulation, thus resulting in chemotherapy resistance.^19^ In addition to chemosensitivity, Wang *et al* proposed that LPCAT3 could cause phospholipid remodelling and promote stem cell proliferation, which is related to colorectal cancer tumorigenesis.^58^ A recent study discovered that LD is a drug reservoir that is responsible for drug depletion in the macrophage of the antibiotics resistance state.^59^

Moreover, although a few previous reports have discussed the roles of LDs in cancers, no study has discussed the potential of targeting LD for cancer therapy. The results of this study revealed that the experimental treatment of LPC with cisplatin did not alter insensitive cells. However, treatment with LLC exhibited excellent growth‐suppressing efficacy. The possible mechanism is as follows: the LPC‐liposome structure (which is a LPC‐cholesterol mixture) can mimic LD structure and then fuse with the LDs to remodel their lipid composition. There is great interest in exploring this possibility further for developing new drug delivery methods. Moreover, this result suggests the great potential of targeting LDs for cancer therapy. The concept of nanodroplet ‘adiposome’^60^ was proposed as a tool for drug delivery, although this concept requires further validation.

In conclusion, here, we stated our novel findings explaining the cellular mechanism of LDLR‐mediated cisplatin insensitivity from the lipid metabolism perspective. We found that LDLR expression confounds platinum chemosensitivity. Moreover, the novel LDLR→LPC→FAM83B→FGFRs regulatory axis revealed through transomics analysis may explain the discrepancies in platinum chemosensitivity.^10^ Finally, LDLR‐altered LD homeostasis contributed to platinum sensitivity, suggesting the potential value of targeting LDs with LLC treatment. Moreover, the lipidome profiling of cancer cells in association with drug sensitivity might be useful for manufacturing drug‐specific liposomes for pharmaceuticals.

## CONFLICT OF INTEREST

The authors of this study declare no conflicts of interest.

## AUTHORS’ CONTRIBUTIONS

L. Chen, WL Ma and WC Cheng conceptualized the study, executed the experiments, conducted bioinformatics analysis and drafted the manuscript. YC Yang, HC Wang and YT Su developed liposome methods; performed bioinformatics analysis, lipid droplet isolation and in vitro experiments; and drafted the methodology section. A. Ahmad assisted to perform animal experiment. YC Hung supported the study partially and edited the manuscript. WL Ma and WC Chang co‐ordinated the research project, supported the project, and edited and approved the final version of the manuscript.

## Data Availability

All data sets can be provided by the corresponding author upon reasonable request.
